# Tension as a key factor in skin responses to pollution

**DOI:** 10.1038/s41598-023-42629-6

**Published:** 2023-09-25

**Authors:** Erika Pambianchi, Zachary Hagenberg, Alessandra Pecorelli, Arianna Pasqui, Jean-Philippe Therrien, Giuseppe Valacchi

**Affiliations:** 1 Department of Animal Science, Plants for Human Health Institute, North Carolina State University, Kannapolis, NC 28081 USA; 2 Department of Biotechnologies, Chemistry and Pharmacy, University of Siena, 53100 Siena, Italy; 3 Toscana Life Sciences Foundation, 53100 Siena, Italy; 4 Department of Food, Bioprocessing and Nutrition Sciences, Plants for Human Health Institute, North Carolina State University, Kannapolis, NC 28081 USA; 5 Department of Environmental Sciences and Prevention, University of Ferrara, 44121 Ferrara, Italy; 6 Department of Food and Nutrition, Kyung Hee University, Seoul, 02447 Korea

**Keywords:** Biological techniques, Environmental sciences, Risk factors

## Abstract

Being the more apparent organ exposed to the outdoor stressors, the effect of pollution on the skin has been widely studied in the last few decades. Although UV light is known as the most aggressive stressor to which our cutaneous tissue is daily exposed, other components of the tropospheric pollution have also shown to affect skin health and functionality. Among them, ozone has been proven to be one of the most toxic due to its high reactivity with the epidermal lipids. Studying the cutaneous effect of pollution in a laboratory setting presents challenges, therefore it becomes critical to employ appropriate and tailored models that aim to answer specific questions. Several skin models are available nowadays: in vitro models (2D cell lines and 3D cutaneous tissues), ex vivo skin explants and in vivo approaches (animals and humans). Although in the last 20 years researchers developed skin models that closely resemble human skin (3D cutaneous tissues), ex vivo skin explants still remain one of the best models to study cutaneous responses. Unfortunately, one important cutaneous property that is not present in the traditional ex vivo human skin explants is the physiological tension, which has been shown to be a cardinal player in skin structure, homeostasis, functional properties and responses to external stimuli. For this reason, in this study, to confirm and further comprehend the harmful mechanism of ozone exposure on the integumentary system, we have performed experiments using the state of art in cutaneous models: the innovative TenSkin™ model in which ex vivo human skin explants are cultured under physiologically relevant tension during the whole experimental procedure. Specifically, we were interested in corroborating previous findings showing that ozone exposure modulates the expression of cutaneous antimicrobial peptides (AMPs). The present work demonstrates that cutaneous exposure to ozone induces AMPs gene and protein levels (CAMP/LL-37, hBD2, hBD3) and that the presence of tension can further modulate their expression. In addition, different responses between tension and non-tension cultured skin were also observed during the evaluation of OxInflammatory markers [cyclooxygenase-2 (COX2), aryl hydrocarbon receptor (AhR), matrix-metallo-proteinase 9 (MMP9) and 4-hydroxy-nonenal (4HNE)]. This current study supports our previous findings confirming the ability of pollution to induce the cutaneous expression of AMPs via redox signaling and corroborates the principle that skin explants are a good and reliable model to study skin responses even though it underlines the need to holistically consider the role of skin tension before extrapolating the data to real life.

## Introduction

Air pollution is now one of the leading causes of premature death, followed distantly by HIV/AIDS infections, parasitic diseases like malaria and other vector-borne infectious ailments^1^. This is in line with the latest estimates of the World Health Organization (WHO) stating that 91% of the urban world’s population breathes polluted air, leading to 4.2 million annual premature deaths^2^.

Environmental pollution is a generic term that comprehends a wide array of compounds present at the ground level. The United State Department of Agriculture (USDA) categorizes them into 6 main groups (based on their chemical and physical characteristics): carbon monoxide (CO), lead (Pb), nitrogen dioxide (NO_2_), ozone (O_3_), particulate matter (PM) and sulfur dioxide (SO_2_)^3^.

Ozone stands among the most dangerous and toxic airborne pollutants for human health^4^, due to its high reactivity with biological membranes.

Around 90% of ozone naturally exists in the stratosphere where it plays a key role in absorbing UV radiation before it reaches the Earth^5^. The anthropogenic-formed ozone is present at the ground-level (troposphere) and it originates from photochemical smog: the reaction between sunlight (UV), nitrogen oxides and hydrocarbons present in car exhaust^6^.

Even though ozone levels can vary based on different conditions (humidity, seasonality, temperature, latitude, altitude), persistently high concentrations have been reported in urban centers, ranging between 0.5 and 0.8 ppm during severe polluted episodes^4,7–9^. Of note, to avoid noxious health effects, the WHO set the threshold of ozone exposure to 0.05 ppm^10^, however, the last reports from WHO itself estimated that 9 out of 10 people breathe air exceeding by far the recommended guidelines^9,10^.

The skin is the second largest organ (after the respiratory tract) to be targeted by ozone exposure.

The mechanism by which ozone induces cutaneous damage is via the direct interaction and oxidation of polyunsaturated fatty acids (PUFAs) and lipids present in the outermost layer of skin, the stratum corneum (SC), leading to the direct generation of aldehydes (i.e., 4-hydroxynonenal-4HNE) and reactive oxygen species (ROS) including H_2_O_2_. All these mediators are able to further carry on ozone toxicity affecting the integrity and functionality of skin barrier as well as perpetuating the damage in deeper cutaneous layers^11–13^.

Indeed, the production of ROS can trigger inflammatory responses propagating the oxidative damage through indirect harmful pathways, such as the activation of pro-inflammatory transcription factors (NFkβ, AP-1, AhR)^14^ and intracellular protein kinases (MAPK, JNK) involved in the degradation of the connective dermal tissue^15^ via the production of metallo-proteinases (MMP9) and the release of pro-inflammatory mediators (COX2, IL-18, IL-1β, etc.).

This crosstalk loop between inflammation and oxidative stress can lead to an aberrant vicious cycle defined as OxInflammation^16^, where oxidative stress that is induced by external stimuli generates and it is fueled by inflammatory responses^17,18^. In addition, it should be mentioned that aryl hydrocarbon receptor (AhR), which is involved in the xenobiotic’s detoxification pathway, can be activated by pollutants such as PMs and O_3_ and even UV^19^. Its activation can further generate ROS production and exacerbate the oxinflammatory tissue responses^20^, giving to this receptor a double edge effect, with a physiological and pathological role in skin.

Our previous in vitro study proposed that the production of pro-inflammatory secondary messengers not only is one of the causes of the development/exacerbation of skin conditions, but it is also the trigger for the production of antimicrobial peptides (AMPs)^21^.

AMPs are small peptides (12–100 amino acid residues), key effectors in innate immune responses^22^; they are encoded by distinct genes, translated from mRNA templates and through the electrostatic interactions and consequent disruption of negatively charged microbial cell membranes^23–25^. AMPs are able to kill a broad spectrum of pathogens without the development of resistance^26,27^.

In humans two main categories have been identified: defensins (the constitutive hBD1 and the inducible hBD2 and hBD3) and cathelicidins (LL-37, encoded by the gene CAMP).

In the skin, the transcription of CAMP can be induced by injury, inflammation and infection^28–30^, leading to the production of the pro-peptide hCAP18, that, once cleaved, releases the mature and active peptide LL-37: a 37 amino acid-long peptide comprising two N-terminal leucines^31^.

Interestingly, increased levels of AMPs have been detected in active lesions of inflammatory skin diseases such as psoriasis and atopic dermatitis^28,32–35^.

Our previous study confirmed the hypothesis that the redox-sensitive upregulation of AMPs is the link between the development of inflammatory skin conditions and the exposure to ozone^21^.

In the context of pollution and skin studies, two approaches can be employed: retrospective studies (i.e. the analysis of the air quality index of a particular period/area and the emergency admissions for cutaneous symptoms collected in that same area and period^36^); or the use of skin models that are directly exposed to pollution followed by endpoint data analysis.

Multiple skin models have been developed and employed over the years, such as in vitro models (2D cell lines and 3D cutaneous models)^37^ and ex vivo skin explants; mostly to replace in vivo approaches that can be expensive and rise ethical concerns (animal testing) or presenting high donor-to-donor variability (human volunteers).

2D cell lines represent the most economical, accessible, reproducible and easy-to-handle cutaneous model available on the market, it can employ immortalized and primary cells however, this monolayer culture of keratinocytes does not undergo terminal differentiation, resulting in the absence of the SC which is the layer mostly affected by ozone exposure^10^.

To overcome this problem 3D models have been developed: they are formed by differentiated keratinocytes able to form the SC, even though not all SC layers are present, making it more accessible to outdoor stressors^37^.

Although a lot of effort and resources has been invested towards the development of 3D cutaneous tissues, one of the best approaches to study cutaneous responses still remain the use of ex vivo human skin explants because, despite the high donor-to-donor variability and the lack of vascular and lymphatic systems, they present all the skin layers, resident immune cells and incorporate multiple skin appendages such as hair follicles and sweat glands (both important to produce AMPs)^21^.

Considering that a perfect model to study skin responses does not exist, cutaneous models need to be chosen depending on the endpoints of interest as well as their advantages and limitations. Based on these premises, 2D cell lines are recommended to evaluate signal transduction pathways and their mechanisms; 3D models and skin explants should be used to assess topical intervention, percutaneous permeation and drug delivery; while feeding studies can be better investigated in animal and clinical interventions.

Unfortunately, all the aforementioned skin models lack an important feature present in human skin which is the physiological tissue tension.

Physiological tension or tensional homeostasis is a mechanical force normally present in healthy skin which is the consequence of the balance between the extracellular forces exerted on skin cells [by both the neighboring cells and the extracellular matrix (ECM)] and the traction forces generated by the cells themselves^38^. Indeed, thanks to the presence of elastic fibers in the dermis, the skin has a degree of mechanical elasticity that maintains a state of constant tension. Skin tension manifests as internal tension-distribution patterns known as Langer’s cleavage lines which are structures fundamental for the maintenance of skin integrity and flexibility^39^.

Tensional homeostasis is important at an organ level as well as at a cellular level since cells can sense tension by being in contact with the actin cytoskeleton and adhesion molecules^40^. Indeed, skin tension has been shown to play cardinal roles in many aspects of skin homeostasis not only during organ morphogenesis but also later in time to maintain skin structure and functions, such as ECM production, proliferative and migratory cellular processes and response to external stimuli^41^.

The importance of cutaneous tension and the need for skin models that closely resemble human skin, gave rise to a new skin model called TenSkin™ (Ten Bio Limited, Dundee, UK, https://ten-bio.com/) that are comprised of human skin cultured under a standardized physiologically relevant tension.

For all these reasons, in the present study we performed the experiments using TenSkin™ and confirmed the hypothesis that ozone exposure induces the upregulation of cutaneous AMPs in tissues with and without tension, reasonably due to oxidative stress (as we previously reported in vitro^21^).

Furthermore, we observed that the presence of tensional homeostasis in skin either anticipated, delayed and/or prolonged the OxInflammatory cutaneous response to ozone compared to non-tension skin exposed to ozone.

These findings corroborate our previous results, confirming the ability of ozone to induce OxInflammatory damage in skin and therefore induce AMPs expression in presence and absence of cutaneous tension. Moreover, these results bring new insights about the importance of skin tension when investigating the effects of environmental stressors that interact and affect skin surface.

## Materials and methods

### Ten bio ex vivo human skin biopsies and ozone (O_3_) exposure

Ex vivo human skin explants, coming from 3 different healthy subjects who underwent elective abdominoplasties, were purchased from Ten Bio. Half of them were cultured under physiologically relevant tension (tension skin, Ten), while the other half was cultured without tension (non-tension skin, NT) (Ten Bio https://ten-bio.com/). Skin models, were then transferred into 6 well plates pre-filled with standard medium provided by the company, using sterile technique. The plates were incubated, for overnight recovery, at 37 °C, 5% CO_2_/95% air atmosphere. All methods were carried out in accordance with relevant guidelines and regulations.

On the following morning the skin explants were placed in semi-solid shipping media provided by the company. Following, half of the Ten Bio models (cultured in presence and in absence of tension) were placed in a plexiglass box connected to the ozone generator (ECO3 model CUV-01, Torino, Italy, Model 306 Ozone Calibration Source, 2B Technologies, Ozone Solution), as previously described (35), and exposed for 4 h at the dose 0.4 ppm.

Skin samples were then collected at 0 h (T0), 3 h (T3), 6 h (T6) and after 24 h upon ozone exposure (T24), for protein, RNA and immunohistochemical analysis.

### RNA extraction and quantitative real time PCR

First, Ten Bio skin explants were homogenized using TRIzol (15596026, Invitrogen) with the help of a tissue homogenizer (Precellys 24 homogenizer, 10 cycles 6500 rpm 3 × 30 s, at 4 °C) and RNA was then isolated using phenol–chloroform extraction method, using a modified version of the protocol described previously by Toni et al.^43^.

cDNA was generated from 1 µg of RNA, via reverse-transcription, using iScript cDNA Synthesis kit (1708841, BioRad), according to the manufacturer's protocol. To investigate transcription levels of AMPs, quantitative real-time PCR was assessed, using SsoAdvanced Universal SYBR Green Supermix (1725271, BioRad), according to the manufacturer’s instructions on a LightCycler 480 machine (Roche).

Gene expression was quantified as the number of cycles needed to reach a threshold value in the intensity of the PCR signal (CT value). Glyceraldehyde-3-phosphate (GAPDH) was employed as a housekeeping gene to normalize, and control samples were used as internal calibrators. After normalization, quantitative relative gene expression, expressed in fold changes, was calculated using the 2^−ΔΔCt^ method.

The primers used are listed here: GAPDH (forward TCGGAGTCAACGGATTTGGT/reverse TTCCCGTTCTCAGCCTTGAC), CAMP (forward CGGTGTATGGGGACAGTGAC/reverse TGGGTACAAGATTCCGCAAA), hBD2 (forward GCATTGCACCCAATACCAGT/reverse CCAAAAACACCTGGAAGAGGCA), hBD3 (forward TATCTTCTGTTTGCTTTGCTCTTCC/reverse CGCCTCTGACTCTGCAATAA).

### Hematoxylin and Eosin (H&E) staining

After collection of the tissue samples, skin explants were fixed in 10% neutral buffered formalin for 48 h at 4 °C, then dehydrated using increasing alcohol gradients, followed by immersion in xylene and paraffin embedding.

For H&E observation, 4 µm thick sections were deparaffinized in xylene and then rehydrated in decreasing alcohol gradients and then stained with Mayer’s hematoxylin solution for 10 min (26043-06, Electron Microscopy Sciences). After rinsing the section in tap water for 15 min, sections were stained with aqueous Eosin Y solution (786-1072, Biosciences) for 3 min and then dehydrated in increasing alcohol solutions, terminating with xylene step. Following, the sections were mounted onto glass slides using a toluene-based solution (SP15-100, Fisher Chemical) and images were taken with (EVOS FL Auto, Life Technologies)^43^.

### Immunohistochemical analysis

For histological observations, 4 µm thick sections were first deparaffinized on a hot plate (60 °C for 30 min) and then in xylene, following which they were rehydrated in decreasing alcohol gradients. Antigen retrieval was achieved via heat-based epitope retrieval with 10 mM sodium citrate buffer (AP-9003-500, Thermo), pH 6.0 with 0.05% Tween 20, at a sub-boiling temperature in a water bath set at 95 °C for 8 min. After cooling for 20 min, the sections were washed 2 X 5 min with 1% Phosphate Buffered Saline (PBS) and then blocked with 5% Bovine Serum Albumin (BSA) in PBS for 45 min. After blocking, sections were then incubated overnight with primary antibodies prepared in 2% BSA/PBS for the following markers: LL-37 (sc-166770, Santa Cruz, dilution 1:50), hBD2 (ab63982, AbCam, dilution 1:500), hBD3 (sc-59495, Santa Cruz, dilution 1:50), 4HNE (AB5605, Millipore, dilution 1:500), AhR (NB 100–128, Novus, 1:150), COX2 (NB 100–868, Novus, 10 µg/ml), MMP9 (NBP2-13173, Novus, 1:200). The next day, sections were washed with 1% PBS 3 X 5 min, followed by 1 h incubation at room temperature with fluorochrome-conjugated secondary antibodies diluted 1:500 in 2% BSA/PBS (AlexaFluor 568 A11004, AlexaFluor 568 A11057, AlexaFluor 488 A11055, AlexaFluor 488 A11008 Invitrogen).

Following, the sections were washed with 1% PBS 3 × 5 min, and then nuclei were stained with DAPI (1874814, Invitrogen, dilution 1:20,000) prepared in PBS, for 2 min at room temperature. Finally, sections were washed one last time with 1% PBS 3 × 5 min and then 1 × 5 min in double distilled (DDI) water, then mounted onto glass slides using PermaFluor aqueous mounting media (TA-006-FM, Thermo) and imaged via epifluorescence on a Zeiss LSM10 confocal microscope equipped at 40× magnification. Quantification of at least 4 images per each condition was performed using ImageJ software^44^.

### Statistical analysis

Each of the variables tested is expressed as mean (in arbitrary units) ± standard deviation (for immunohistochemical analysis) or ± standard error (for qRT-PCR analysis).

PCR data is representative of the average of 3 donors while immunofluorescence staining analysis is representative of 1 donor in triplicate.

Within each timepoint, the condition of non-tension skin exposed to ozone (O3 NT) was compared to the respective non-tension skin exposed to air (Air NT), similarly, the condition of tension skin exposed to ozone (O3 Ten) was compared to the respective tension skin exposed to air (Air Ten).

Statistical analyses were performed via GraphPad Prism 6 software (GraphPad Software Inc.).

The differences between groups were evaluated by analysis of variance (ANOVA) considering the timepoints (T0, T3, T6, T24) separately, followed by Tukey’s post-hoc test. A p-value < 0.05 was considered statistically significant.

## Results

### Ozone exposure increases LL-37 levels in human skin biopsies in presence and absence of tension

**Figure 1:**
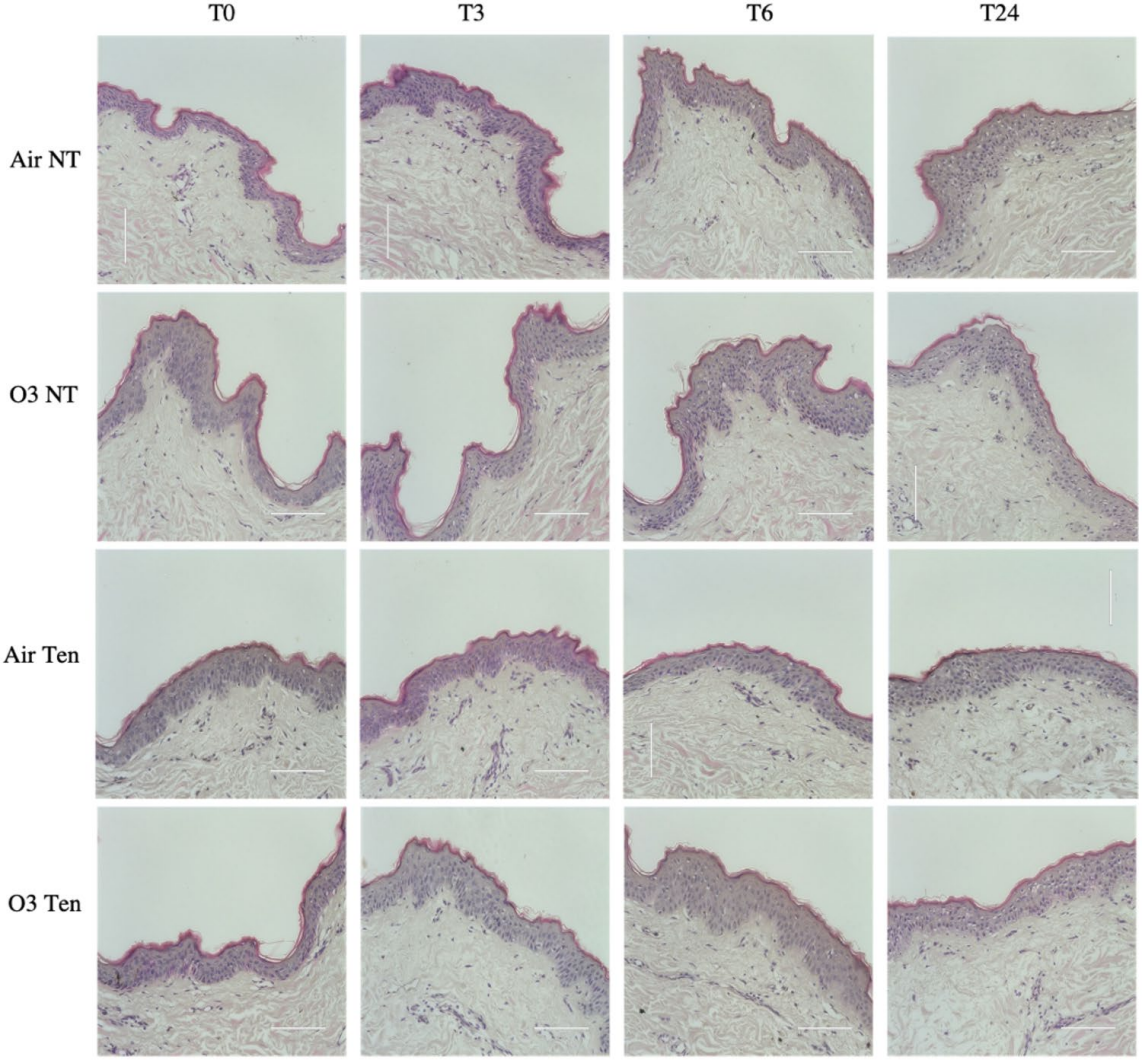
Presence or absence of tension and ozone exposure does not alter tissue morphology. Hematoxylin and Eosin (H&E) staining of Ten Bio ex vivo human skin biopsies cultured in presence (tension—Ten) or in absence (non-tension—NT) of tension and exposed to 4 h of either air (Air) or ozone (O3) at 0.4 ppm. Samples were collected directly after exposure (T0), after 3 h (T3), 6 h (T6) and 24 h (T24). Original magnification ×40, scale bar 100 µm.

First, we confirmed that the chosen concentration of ozone (0.4 ppm, 4 h) and the experimental settings did not cause any morphological alterations in Ten Bio models cultured in presence or in absence of tension, suggesting that the conditions used during the experimental procedure and the dose of ozone used are not overly aggressive (Fig. 1).

To further validate our previous data obtained in in vitro*, *in vivo and ex vivo skin models^16^, we first evaluated the cutaneous expression of AMPs upon ozone exposure in presence and absence of tension.

**Figure 2:**
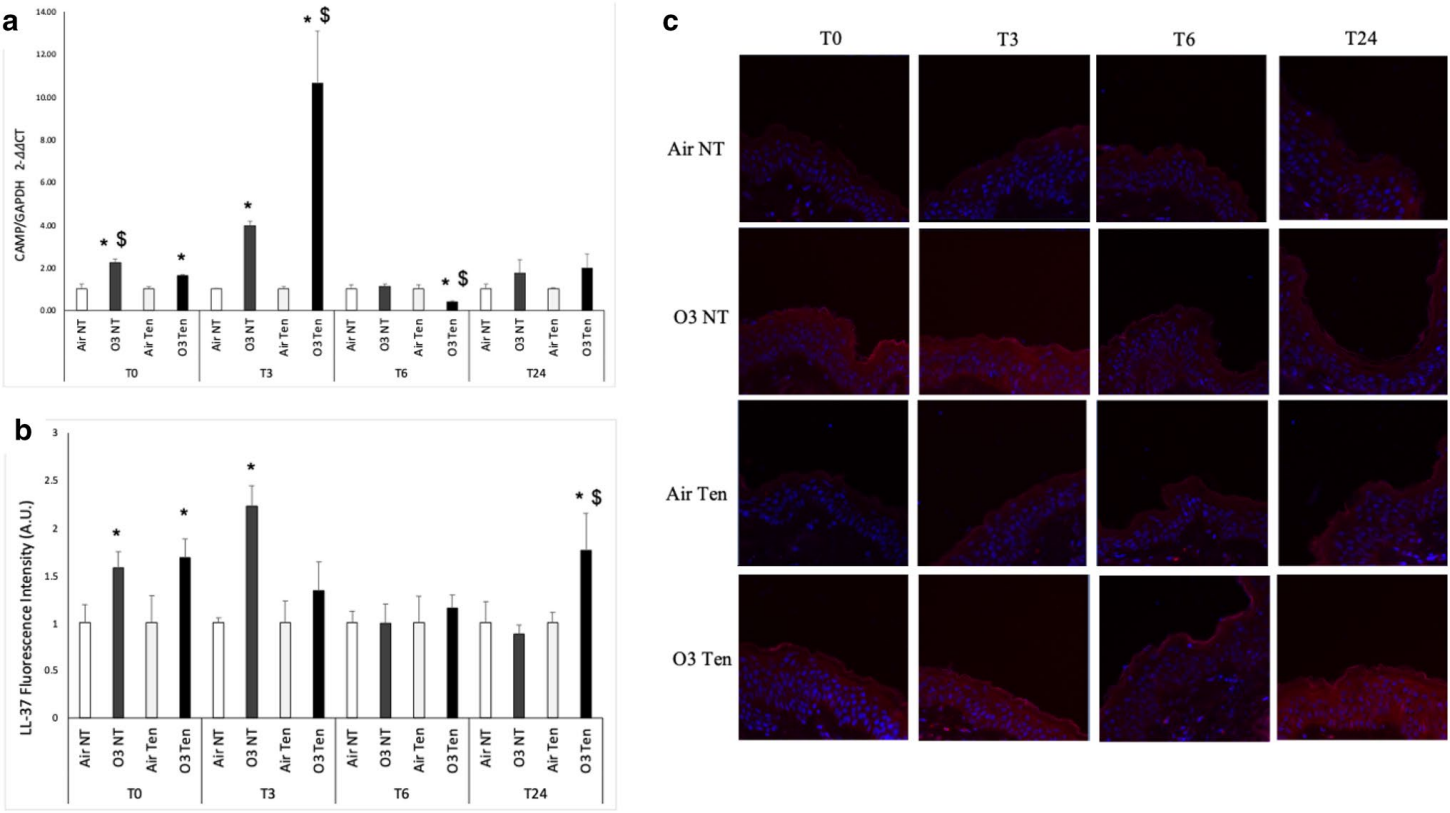
Transcripts and protein levels of CAMP/LL-37 are increased upon ozone exposure and the presence of tension modulates their expression. Ex vivo human skin biopsies were cultured with (tension—Ten) or without (non-tension—NT) tension (Ten Bio models) and then exposed for 4 h to either air or 0.4 ppm of O_3_. Samples were collected directly after exposure (T0), after 3 h (T3), 6 h (T6) and 24 h (T24). (**a**) qRT-PCR analysis of transcripts levels of CAMP. (**b**) Semi-quantification of the LL-37 immunofluorescence intensities performed by ImageJ. (**c**) Levels of LL-37, red fluorescence staining represents LL-37 immunoreactivity, while cell nuclei are stained in blue with DAPI. Original magnification ×40. Results are expressed as arbitrary units ± standard deviation (IF) or standard error (qRT-PCR). *p-value < 0.05 by ANOVA.

As shown in Fig. 2a ozone exposure, in presence and absence of tension, affected the cutaneous transcript levels of the gene CAMP: while both non-tension skin and tension-skin responded to ozone exposure at T0 at T3 with an increase in CAMP transcripts, after 3 h (T3) the extent of the gene expression increase in CAMP mRNA levels was strikingly different: 10-folds increase in tension-skin compared to 4-folds increase in non-tension skin (Fig. 2a).

In addition, as depicted in Fig. 2b, ozone exposure induced a significant increase in LL-37 protein levels in skin tissues compared to air, in both presence and absence of tension at T0. Of note, this upregulation of LL-37 protein levels was still present in non-tension skin after 3 h (T3) but clearly disappeared after 6 (T6) and 24 h (T24) post ozone exposure. On the other hand, the LL-37 protein levels in ozone-exposed tension-skin, although not increased at T3 and T6, were significantly upregulated at a later time point (T24) (Fig. 2b,c).

### Effect of tension on cutaneous hBD2 levels upon ozone exposure

**Figure 3:**
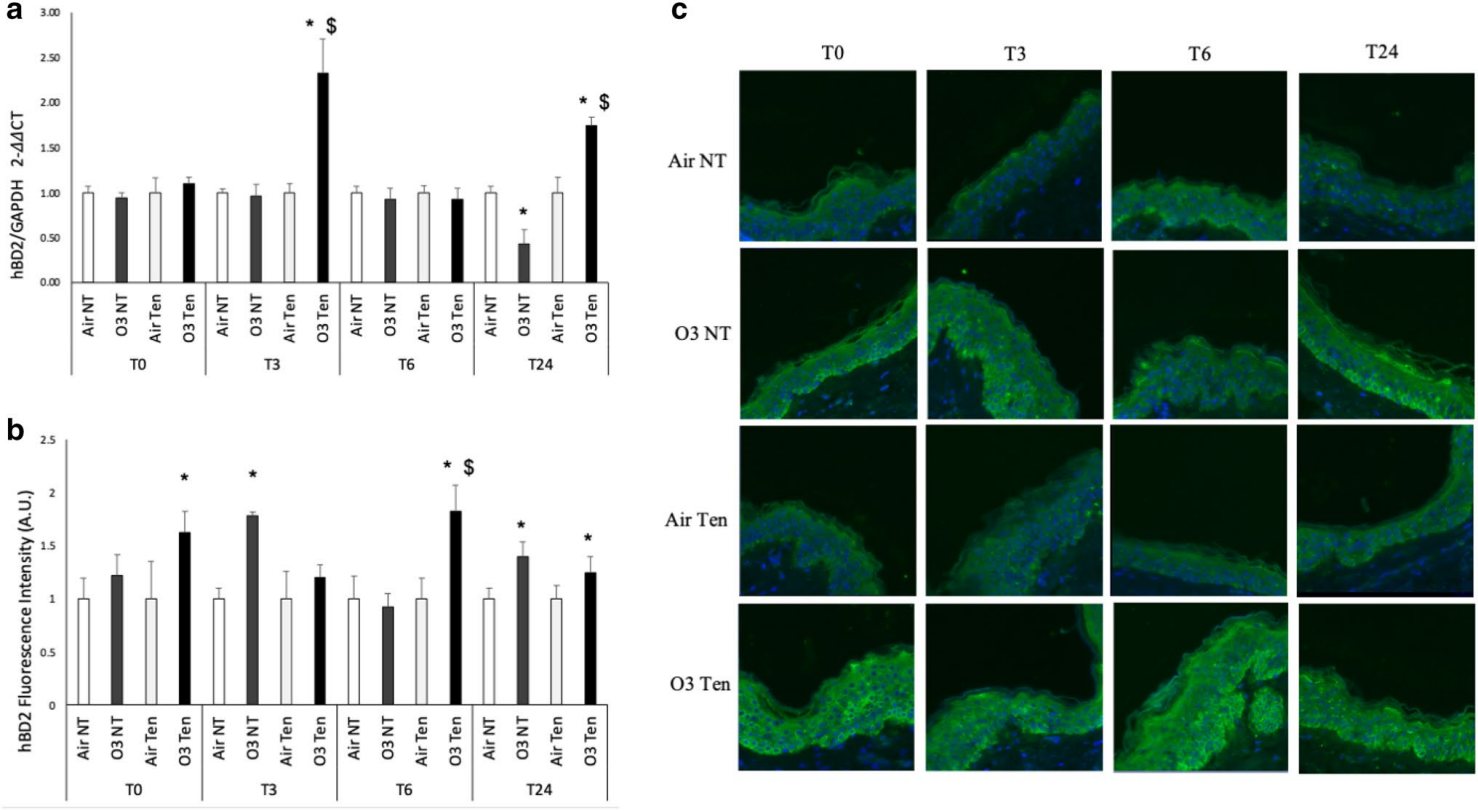
Transcripts and protein levels of hBD2 are increased upon ozone exposure and the presence of tension differently modulates their expression. Ex vivo human skin biopsies were cultured with (tension—Ten) or without (non-tension—NT) tension (Ten Bio models) and then exposed for 4 h to either air or 0.4 ppm of O_3_. Samples were collected directly after exposure (T0), after 3 h (T3), 6 h (T6) and 24 h (T24). (**a**) qRT-PCR analysis of transcripts levels of hBD2. (**b**) Semi-quantification of the hBD2 immunofluorescence intensities performed by ImageJ. (**c**) Levels of hBD2, green fluorescence staining represents hBD2 immunoreactivity, while cell nuclei are stained in blue with DAPI. Original magnification ×40. Results are expressed as arbitrary units ± standard deviation (IF) or standard error (qRT-PCR). *p-value < 0.05 by ANOVA.

As shown in Fig. 3, ozone exposure clearly induced the levels of hBD2 in the stratum basale layer of both tension and non-tension skin models, and also in this case the presence of tension affected the timing of hBD2 levels increase. As depicted in Fig. 3a, ozone induced hBD2 transcript levels only in tension skin at T6 and T24 compared to its control air and to the non-tension skin exposed to ozone (Fig. 3a), confirming that tension plays an important role in skin responses.

In addition, as shown in Fig. 3b,c, right after O_3_ exposure (T0) only tension-skin showed a significant increase in hBD2 protein levels after ozone exposure compared to control air.

Interestingly, at T3 this trend between tension and non-tension skin response to ozone was reverted. In addition, 6 h after ozone exposure (T6), we observed a second wave of hBD2 upregulation in tension skin exposed to ozone compared to its respective air and also to O_3_ exposed non-tension skin. Surprisingly, at T24, we detected upregulated protein levels in both tension and non-tension skin exposed to ozone compared to their respective air controls (Fig. 3b,c).

### Effect of tension on cutaneous hBD3 levels upon ozone exposure

To obtain a holistic picture of the cutaneous AMPs levels upon ozone exposure in presence or not of tension we further evaluated the hBD3 levels.

**Figure 4:**
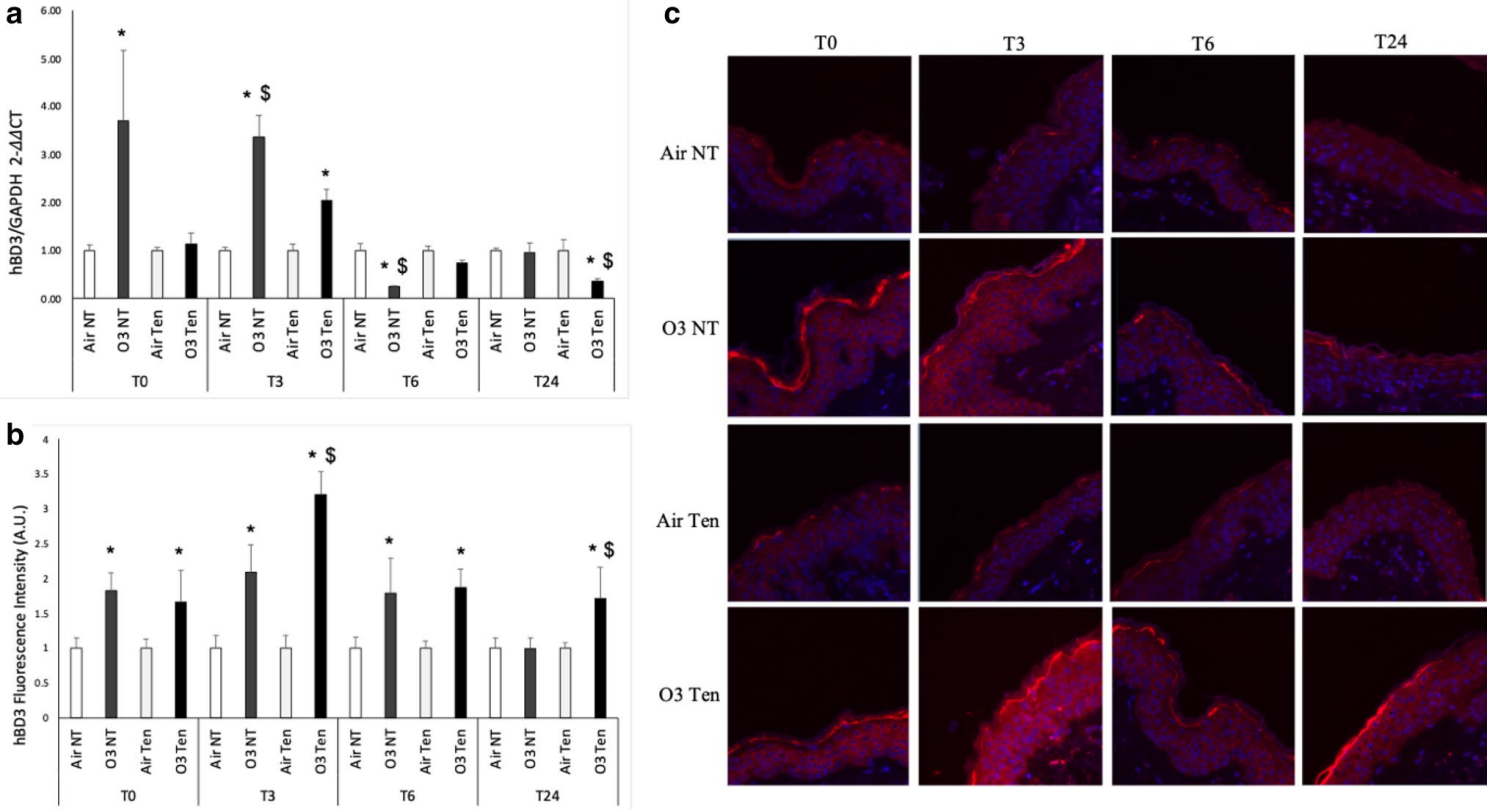
Transcripts and protein levels of hBD3 are increased upon ozone exposure and the presence of physiological tension differently modulates their expression. Ex vivo human skin biopsies were cultured with (tension—Ten) or without (non-tension—NT) tension (Ten Bio models) and then exposed for 4 h to either air or 0.4 ppm of O_3_. Samples were collected directly after exposure (T0), after 3 h (T3), 6 h (T6) and 24 h (T24). (**a**) qRT-PCR analysis of transcripts levels of hBD3. (**b**) Semi-quantification of the hBD3 immunofluorescence intensities performed by ImageJ. (**c**) Levels of hBD3, red fluorescence staining represents hBD3 immunoreactivity, while cell nuclei are stained in blue with DAPI. Original magnification ×40. Results are expressed as arbitrary units ± standard deviation (IF) or standard error (qRT-PCR). *p-value < 0.05 by ANOVA.

As shown in Fig. 4a, we detected significantly higher hBD3 mRNA levels in non-tension skin exposed to ozone at T0. At 3 h post ozone exposure, both tension skin and non-tension skin responded with increased hBD3 transcript levels compared to their corresponding air controls and we observed a higher increase in non-tension skin compared to tension-skin (Fig. 4a).

As shown in Fig. 4b,c, ozone exposure induced increased hBD3 protein levels in both tension and non-tension skin models, compared to air at T0 and at T6. Of note, after 3 h tension skin exposed to ozone showed a significantly higher upregulation of hBD3 levels in the upper epidermal layers compared to non-tension skin and to the respective air conditions (Fig. 4b,c).

At a later time (T24), only tension skin was able to respond to O_3_ exposure with a significant hBD3 upregulation compared to its respective air and also to non-tension skin (Fig. 4b,c).

These results corroborate our previous findings observed in 2D, ex vivo human skin biopsies and in vivo (forearms of human volunteers) models^21^ confirming that ozone exposure clearly and significantly increases AMPs expression at both protein and gene levels, besides the different time regulation due to the presence or absence of tensional homeostasis.

### Oxidative stress is associated with cutaneous AMPs regulation

To further understand the mechanisms by which ozone increases AMPs levels when tension is a variable, we decided to evaluate OxInflammatory markers involved in redox-signaling^45–47^.

**Figure 5:**
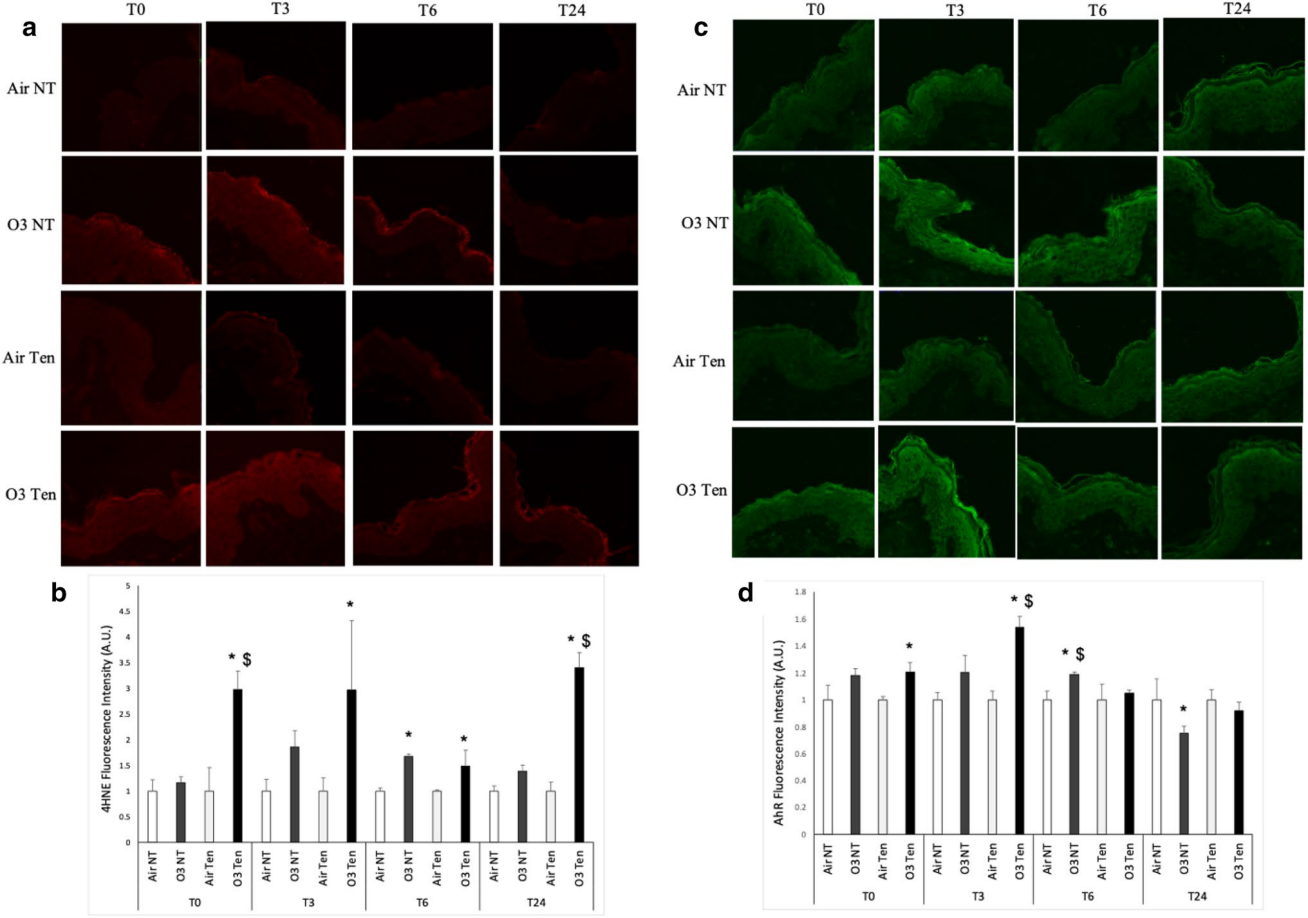
Ozone exposure upregulates protein levels of 4HNE and AhR, while the presence of tension affects the timing of the response. Ex vivo human skin biopsies were cultured with (tension—Ten) or without (non-tension—NT) tension (Ten Bio models) and then exposed for 4 h to either air or 0.4 ppm of O_3_. Samples were collected directly after exposure (T0), after 3 h (T3), 6 h (T6) and 24 h (T24). (**a**) Levels of 4HNE, red fluorescence staining represents 4HNE. Original magnification ×40. (**b**) Semi-quantification of the 4HNE immunofluorescence intensities performed by ImageJ. (**c**) Levels of AhR, green fluorescence staining represents AhR immunoreactivity. Original magnification ×40. (**d**) Semi-quantification of the AhR immunofluorescence intensities performed by ImageJ. Results are expressed as arbitrary units ± standard deviation. *p-value < 0.05 by ANOVA.

As shown in Fig. 5, ozone exposure induces increased levels of 4HNE in tension skin at all time points evaluated (T0, T3, T6, T24) compared to air. Interestingly, these levels are also significantly higher compared to non-tension skin exposed to ozone at T0 and T24. Although trends in increase of the 4-HNE levels are present upon ozone exposure in non-tension skin, they are significantly higher than the corresponding air control only at T6 (Fig. 5a,b).

A similar trend is visible in Fig. 5c,d where it is shown that the levels of AhR are augmented upon ozone exposure at T0 and T3, only in tension skin exposed to ozone compared to its respective air and to non-tension skin (T3). A delayed response (T6) was detected in non-tension skin exposed to ozone which induced a significant increase in AhR protein levels compared to both non-tension air and tension skin exposed to the pollutant. At this stage, we observed no clear differences between AhR levels in both models exposed to either ozone or air after 24 h (Fig. 5c,d).

**Figure 6:**
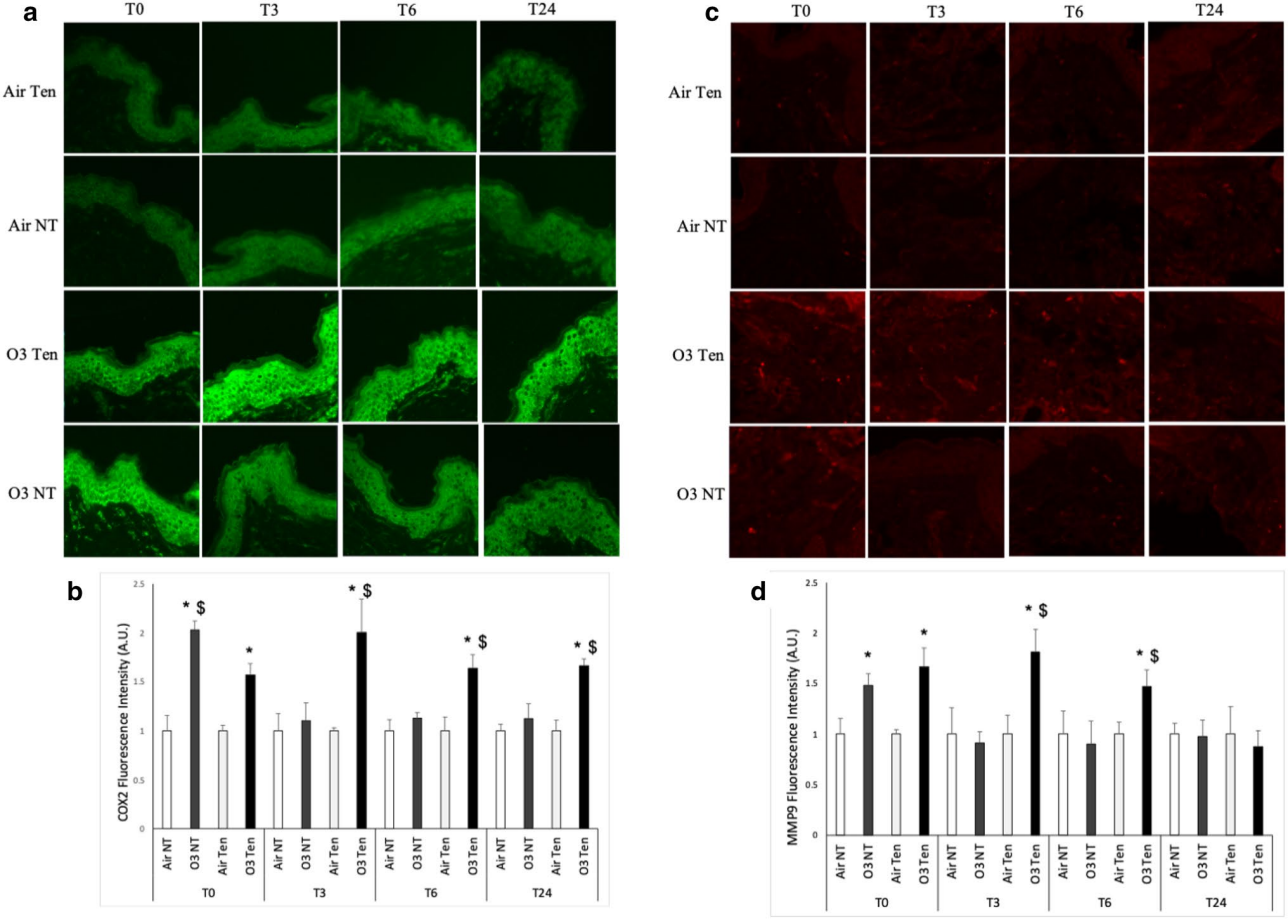
Ozone exposure upregulates protein levels of COX2 and MMP9, while the presence of tension affects the timing of the response. Ex vivo human skin biopsies were cultured with (tension—Ten) or without (non-tension—NT) tension (Ten Bio models) and then exposed for 4 h to either air or 0.4 ppm of O_3_. Samples were collected directly after exposure (T0), after 3 h (T3), 6 h (T6) and 24 h (T24). (**a**) Levels of COX2, green fluorescence staining represents COX2 immunoreactivity. Original magnification ×40. (**b**) Semi-quantification of the COX2 immunofluorescence intensities performed by ImageJ. (**c**) Levels of MMP9, red fluorescence staining represents MMP9 immunoreactivity. Original magnification ×40. (**d**) Semi-quantification of the MMP9 immunofluorescence intensities performed by ImageJ. Results are expressed as arbitrary units ± standard deviation. *p-value < 0.05 by ANOVA.

Moreover, as depicted in Fig. 6a,b, the exposure of Ten Bio models to ozone induced, at T0, increased cyclooxygenase-2 (COX2) protein levels in both tension and non-tension skin compared to air conditions; of note, the levels of COX2 protein expression in non-tension skin exposed to ozone were significantly higher than the ones in tension skin exposed to O_3_.

As for all the other timepoints (T3, T6, T24), ozone exposure induced a clear upregulation of COX2 levels solely in tension skin upon ozone exposure compared to all the other conditions evaluated (tension skin exposed to air and non-tension skin exposed to ozone). Surprisingly, tension skin was able to respond to ozone exposure by inducing the levels of COX2, even after 24 h from ozone exposure (Fig. 6a,b).

A similar trend was visible also for metallo-proteinase-9 (MMP9). Indeed, as shown in Fig. 6c,d, a clear increment in MMP9 levels was noticed right after ozone exposure (T0) in both tension and non-tension skin models exposed to ozone, compared to the respective air conditions.

However, as already seen for COX2 levels, after 3 and 6 h from ozone exposure (T3 and T6), only the skin model that was cultured under physiologically relevant tension showed continuously induced MMP9 protein levels compared to air and to non-tension skin exposed to ozone, and this increment was lost 24 h post-exposure in all the examined conditions (Fig. 6c,d).

## Discussion

Exposure to environmental pollutants is inevitable and it has been estimated to decrease human life span by 10–15 years^48^. In particular, recent reports evidenced that ozone levels are still rising, making the exposure to this pollutant even more of a concern for future generations^49^.

Ozone exposure has been associated not only with the triggering of new inflammatory skin conditions and the worsening of pre-existing ones (such as atopic dermatitis, psoriasis, acne^50–53^) but also with the increase of AMPs levels^21^.

AMPs constitute a ubiquitous and important component of innate immunity by protecting our bodies against pathogen infiltration. New evidences, moreover, found them capable to act as anti-inflammatory molecules in different circumstances^54^, indeed, new cosmeceutical formulations targeting skin aging, list antimicrobial peptides within their ingredients to ameliorate cutaneous functions and structure^55,56^.

However, several studies suggested a potential negative involvement of AMPs in the pathogenesis of multiple skin conditions like atopic dermatitis, psoriasis, acne vulgaris, rosacea, systemic lupus erythematosus and systemic sclerosis^28,32–35^. Therefore, AMPs induction could represent a double-edge sword: a friend that assists in skin immunity and a foe that contributes to inflammatory skin pathologies^56^.

Altered AMPs levels have been recently observed upon different pollutants exposure (ozone, UV, particulate matter^57–62^) however it is currently unclear if this response to pollution inhibits or promotes cutaneous inflammation.

We believe that the physiological rationale behind AMPs upregulation after ozone exposure could be explained in 2 ways. First, it could be interpreted as a non-specific response of skin to external stimuli and secondly, AMPs could be triggered presumably to activate an antibacterial response to prevent secondary infections caused by pollution that, by impairing skin structure, makes it more exposed to the entry of other pollutants and pathogens.

In fact, the structural epidermal damage induced by exposure to pollutants not only induces a general impairment of skin barrier function and consequent excessive trans epidermal water loss (TEWL) but it can also favor the entry of pathogens and facilitate the access of PM via hair follicles and trans-dermally, allowing them to reach the dermis contributing to systemic damage^63–65^.

Even though the purpose of AMPs increase upon ozone exposure is still unclear, we have identified that the mechanism of its occurrence is via redox regulation^21^. In fact, as we previously demonstrated and here confirmed, ozone, via lipid peroxidation of cutaneous lipids, induces the formation of oxidative mediators (4HNE and H_2_O_2_) that activate pro-inflammatory transcription factors (NFkβ, AP-1, AhR)^14^ and intracellular protein kinases (MAPK, JNK) leading to the release of inflammatory cytokines like COX2, IL-18, IL-1β^66^, and the cleavage of inactive metallo-protease zymogens (pro-MMP9) into active ones (MMP9) able to degrade the extracellular matrix components^15^.

Increased levels of 4HNE, AhR, COX2 and other inflammatory markers have been found elevated and involved in the pathogenesis of chronic skin conditions^67–69^, while augmented levels of matrix MMP9 and reduced levels of collagen and elastin are known to be hallmarks of skin aging^70^.

On the other hand, the activation of pro-inflammatory transcription factors has also been associated with the triggering of AMPs, since AMPs promoters present multiple binding sites for NFkβ, AP-1 and AhR^71,72^.

In fact, in silico analysis brought to light the presence of multiple binding sites for both NFkβ and AP-1 in hBD2 promoter^71,72^ and three different sites for AhR, in hBD3 promoter^21^. This is in line with a recent study from 2019 showing that the levels of hBD3 transcripts, induced in the first place by the cutaneous infection with *Staphylococcus epidermidis*, were reduced after the blocking of AhR signaling^73^.

To confirm the theory that oxidative stress regulates AMPs transcription, in our previous work we showed that, in human keratinocytes, H_2_O_2_ and 4HNE, originating from the interaction of ozone with the PUFAs present in the stratum corneum, were responsible mediators of the effect of ozone in upregulating cutaneous AMPs levels^21^.

The correlation between AMPs and markers of oxidative stress has become clearer in the present work, as we were able to show increased transcript and protein levels for all the AMPs evaluated (CAMP/LL-37, hBD2, hBD3) upon ozone exposure in both tissues cultured with and without tensional homeostasis, corroborating the previous findings^21^. The induction of the transcript and protein levels of antimicrobial peptides after ozone exposure, could be explained by the activation of the redox-sensitive transcription factors NFkβ and AP-1, confirmed moreover by the increased levels of COX2 (that is under the regulation of NFkβ)^74^ and MMP9 which instead depends on AP-1 activation^75^.

In this current work we were not just interested in confirming previous hypotheses, we also wanted to investigate the possible role that skin tension plays in the cutaneous responses to ozone.

To test this, we used Ten Bio models that mimic the cutaneous physiological tension, and observed that the tension was able to modulate the timing and the degree of the response to ozone.

This effect could be explained by multiple factors: firstly, the reduction of the micro folds in skin cultured under tension increases the unit of surface area exposed to ozone, mimicking real-life cutaneous exposure. Secondly, as previously showed in 3D in-vitro skin models, the presence of tensional homeostasis improves the cutaneous physiological functions of the model, including fibroblasts ECM production, keratinocytes proliferation and greater responsiveness of cells to function molecules, possibly extending the longevity and functionality of this tension skin model^41^.

We theorize that this more acute response of skin tension exposed to ozone, caused an earlier formation of ozone mediators, compared to non-tension skin which, consequently, induced an earlier activation of transcription factors and earlier formation of the OxInflammatory response. We occasionally observed an apparently anticipated increase (T0, T3) of some protein levels (hBD3, COX2) in non-tension skin compared to tension skin upon ozone exposure, however, this could be explained by the possibly even earlier response (during the 4 h of ozone exposure) of tension skin, unfortunately missed in the earliest time points. Indeed, we suggest that the activation of these pathways in non-tension skin was triggered right after (and not during) ozone exposure allowing us to detect increased protein levels at T0 and T3, compared to tension skin. We believe that shorter exposures to ozone could allow us to observe an anticipated response of tension skin compared to non-tension skin.

This work validates the knowledge that the use of skin biopsies, even if not cultured under tension and although lacking some integumentary features (e.g., blood and lymphatic vessels important for distribution and metabolization of compounds that can impact cellular responses like antioxidants and hormones) is still one of the best and complete models to generate hypotheses and study skin responses.

Of course, it is always recommended to perform experiments knowing the limitations of each model.

Furthermore, these findings expose the importance of considering the tension of skin when investigating the effects of outdoor stressors that interact with cutaneous tissues. In fact, although the use of Tenskin™ model may not significantly affect the results of studies evaluating substances or aggressors capable of penetrating the skin layers (like UV), the use of skin tension should be an essential feature present when investigating pollutants like ozone and particulate matter that have as a major mechanism of direct damage the interaction with lipids and PUFAs in the stratum corneum.

For these reasons and thanks to all the technological improvements that in the last decade researchers have been able to realize and made available to the field of skin biology, it is fundamental to choose relevant and appropriate skin models based on their limitation and advantages, as well as on the endpoints of interest. For instance, Ten Bio model would be beneficial not only to study pollutants that interact with the stratum corneum but also to perform physiological tests (e.g., drug delivery) and to evaluate specific aspects that closely resemble biological and real-life responses, since cutaneous tensional homeostasis has been proven fundamental for skin structure and functions at many stages of life^32^.

Further work should focus on the investigation of the molecular mechanisms at the base of cutaneous tension responses in the context of pollution-induced oxidative stress and antimicrobial peptides expression, to be able to obtain data that can be reliably extrapolated to real-life.

## Data Availability

The datasets used and/or analyzed during the current study available from the corresponding author on reasonable request.
